# Supplementary material for: Viral syncytia evolve to resist interferon
Source: Nat Commun. 2026 Jul 20;17:8851. doi: 10.1038/s41467-026-74676-8 (PMC13500464; doi:10.1038/s41467-026-74676-8)
Supplement: Supplementary file 2 [file 41467_2026_74676_MOESM2_ESM.pdf]

nature portfolio

Peer Review File

Viral Syncytia Evolve to Resist Interferon

Corresponding Author: Dr Jonathan Yewdell

This manuscript has been previously reviewed at another journal. This document only contains information relating to versions considered at Nature Communications.

This file contains all reviewer reports in order by version, followed by all author rebuttals in order by version.

Version 0:

Reviewer comments:

Reviewer #1

(Remarks to the Author)
The authors addressed all the reviewer's concerns. Now this manuscript is ready for publication.

Reviewer #2

(Remarks to the Author)
The paper is revised with new data and with clarification in the text. Both improve the strength of the study, and the new data directly address reviewer comments. The authors however have side-stepped one of the major comments:

Comment 4:
What is not real clear is how SARS2 might really continue to replicate in the fused syncytia- this is not at all really addressed and is fundamental to the model- if SARS2 can replicate in the fused cells then this is likely a main way that the virus really does avoid IFN and spread through the tissue. How the virus actually replicates and spreads in and from the fused cells needs to be directly addressed with experiments that measure viral replication, subgenomic viral RNA expression, and infectious viral particle production from fused cells and from non-fused cells. The dynamics of this replication in each fused and non-fused cell model then needs to be quantified to reveal how the virus replicates in fused cells. Is this replication substrate then the preferred venue for the virus versus in non-fused cells if indeed IFN is taken out of the picture? Also how does the double membrane vesicle of virion accumulation fit into all of this? These items should be addressed to solidify the conclusions presented by the authors.

Response:
We don't understand the reviewer's point. Cell fusion increases viral capacity to reproduce; each cell providing a new set of ribosomes, membranes etc. to create new virions, which are still released by cells. While virion production is somewhat lower than in non-fused cells in the absence of IFN, it is far higher than in the presence of IFN and therefore, is under positive selection.

Please provide a clear response to each item in this comment, including how fusion of cells impacts double membrane vesicle virus replication/accumulation. Are these vesicles present in fused cells or does the virus build fused cell-specific substrate(s) for replication?

Version 1:

Reviewer comments:

Reviewer #2

(Remarks to the Author)

The authors have addressed my concerns

Open Access This Peer Review File is licensed under a Creative Commons Attribution 4.0 International License, which permits use, sharing, adaptation, distribution and reproduction in any medium or format, as long as you give appropriate credit to the original author(s) and the source, provide a link to the Creative Commons license, and indicate if changes were made.
In cases where reviewers are anonymous, credit should be given to 'Anonymous Referee' and the source.
The images or other third party material in this Peer Review File are included in the article's Creative Commons license, unless indicated otherwise in a credit line to the material. If material is not included in the article's Creative Commons license and your intended use is not permitted by statutory regulation or exceeds the permitted use, you will need to obtain permission directly from the copyright holder.
To view a copy of this license, visit https://creativecommons.org/licenses/by/4.0/

Point-by-point Response to Reviews

We thank the reviewer for their positive feedback and constructive comments on our revised manuscript, "*Viral Syncytia Evolve to Resist Interferon*." Below, we provide our point-by-point response to the latest comments.

Reviewer #2 (Remarks to the Author):

The paper is revised with new data and with clarification in the text. Both improve the strength of the study, and the new data directly address reviewer comments. The authors however have side-stepped one of the major comments:

Comment 4:

What is not real clear is how SARS2 might really continue to replicate in the fused syncytia- this is not at all really addressed and is fundamental to the model- if SARS2 can replicate in the fused cells then this is likely a main way that the virus really does avoid IFN and spread through the tissue. How the virus actually replicates and spreads in and from the fused cells needs to be directly addressed with experiments that measure viral replication, subgenomic viral RNA expression, and infectious viral particle production from fused cells and from non-fused cells. The dynamics of this replication in each fused and non-fused cell model then needs to be quantified to reveal how the virus replicates in fused cells. Is this replication substrate then the preferred venue for the virus versus in non-fused cells if indeed IFN is taken out of the picture? Also how does the double membrane vesicle of virion accumulation fit into all of this? These items should be addressed to solidify the conclusions presented by the authors.

Please provide a clear response to each item in this comment, including how fusion of cells impacts double membrane vesicle virus replication/accumulation. Are these vesicles present in fused cells or does the virus build fused cell-specific substrate(s) for replication?

We thank the reviewer for raising this important point regarding how *SARS-CoV-2* replicates and spreads within fused syncytial cells. In the submitted manuscript, we measured multiple aspects of viral replication, including replication kinetics (Fig. 1), reporter gene expression (Figs. 1–3), subgenomic viral RNA levels (Fig. 4), and infectious viral particle production (Figs. 1–4) across cell lines, air–liquid interface cultures, and animal models. These analyses were performed under conditions that include both syncytia-forming and non-syncytia-forming infections, as the reviewer stipulated. Across all systems, our data consistently support the conclusion that in the presence of interferon (IFN), syncytia-forming viruses exhibit a clear replication and dissemination advantage.

Our functional experiments further define the basis for this advantage. IFN does not differentially inhibit initial infection by syncytia-forming versus non-syncytia-forming viruses; rather, it selectively restricts cell-free spread. In contrast, syncytia-mediated infection proceeds efficiently under IFN conditions, enabling continued viral gene expression and expansion of infection foci. Importantly, emetine-block experiments demonstrate that viral gene expression in syncytia is largely supported by newly fused recipient cells, indicating that syncytia provide access to

additional cellular resources that sustain infection despite antiviral pressure. Together, these findings establish syncytia as a functionally distinct and IFN-resistant mode of viral propagation.

To directly address the reviewer's question regarding virion assembly and intracellular accumulation, we performed transmission electron microscopy (TEM) analyses comparing fused and non-fused infected cells. We compared a non-syncytia-forming variant (R685S) with a strongly syncytia-forming variant (Delta-Spike) following 24-hour infection in Vero-TMPRSS2 cells.

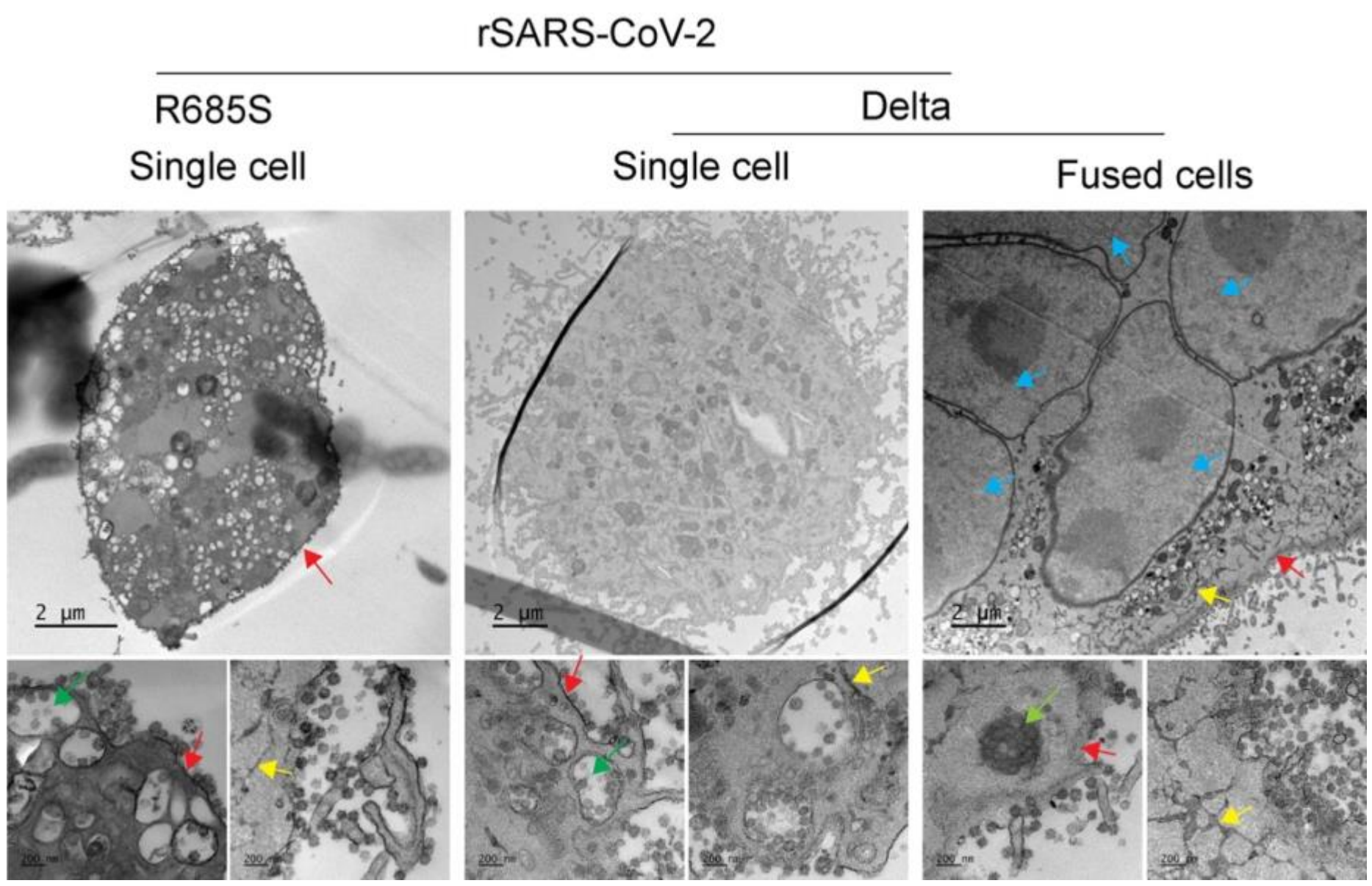

In non-fused infected cells, both variants show abundant virion accumulation within double-membrane vesicles (DMVs) (green arrows), consistent with canonical coronavirus replication structures. In contrast, fused cells (blue arrows) display a marked reduction in morphologically typical DMVs. Instead, virion particles are more frequently observed in regions that lack clearly defined surrounding membranes (light green arrows). In addition, fused cells exhibit pronounced plasma membrane disruption (red arrows) and accumulation of membrane-bound organelles, likely derived from the secretory pathway, near the cell periphery (yellow arrows). These changes collectively indicate substantial reorganization of intracellular membrane architecture in syncytia. They are consistent with our observation that lipid-synthesis-related mRNAs are upregulated in syncytia (included in our last rebuttal, but not in the current manuscript).

While TEM does not directly identify sites of viral RNA synthesis or assembly, the consistent reduction in classical DMV structures together with the altered distribution of virion-like particles suggests that canonical SARS-CoV2 membrane reorganization is disrupted in syncytia. When considered alongside our functional data, these observations suggest that syncytia support viral replication and assembly in a cellular context distinct from that of non-fused cells. We note that the reduced prevalence of morphologically typical DMVs in syncytia likely reflects the fundamentally altered cellular architecture of multinucleated cells, including increased cytoplasmic volume, disruption of ER organization, and extensive membrane remodeling. In this

context, canonical DMV structures may be less abundant, more dispersed, or less readily identifiable by TEM, even as viral gene expression and replication proceed efficiently.

These TEM observations provide preliminary insight into differences in membrane organization and virion localization between fused and non-fused cells. However, they also raise mechanistic questions about the precise sites of viral RNA synthesis and assembly that cannot be resolved solely by TEM. Fully addressing these questions would require substantial, technically demanding additional experiments (e.g., immuno-EM or correlative imaging) beyond the scope of the current study. To avoid overinterpretation and ensure that the conclusions of the manuscript remain fully supported by definitive evidence, we have therefore not included these TEM data in the main manuscript. We would, however, be happy to include them if the reviewer and editor feel that their inclusion would strengthen the study.
