# Supplementary material for: Viral syncytia evolve to resist interferon
Source: Nat Commun. 2026 Jul 20;17:8851. doi: 10.1038/s41467-026-74676-8 (PMC13500464; doi:10.1038/s41467-026-74676-8)
Supplement: Supplementary file 3 [file 41467_2026_74676_MOESM3_ESM.pdf]

Description of Additional Supplementary Files

File Name: Supplementary Data 1
Description: NGS sequencing result of viral stocks

File Name: Supplementary Movie 1
Description: Vero cells were infected by rVSV-S wt at a MOI of 0.01. After virus inoculation, media were replaced with fresh media. Video record was recorded 16 hpi for 20h.

File Name: Supplementary Movie 2
Description: Vero cells were treated with IFN-β at 0.05 ng/ml for 20 h prior to infection by rVSV-S wt at a MOI of 0.01. After virus inoculation, media were replaced with fresh media containing the same amount of IFN-β. Video record was recorded 16 hpi for 20h.

File Name: Supplementary Movie 3
Description: Vero cells were infected by rVSV-S R685S at a MOI of 0.01. After virus inoculation, media were replaced with fresh media. Video record was recorded 16 hpi for 20h.

File Name: Supplementary Movie 4
Description: Vero cells were treated with IFN-β at 0.05 ng/ml for 20 h prior to infection by rVSV-S R685S at a MOI of 0.01. After virus inoculation, media were replaced with fresh media containing the same amount of IFN-β. Video record was recorded 16 hpi for 20h.
