# Supplementary material for: Viral syncytia evolve to resist interferon
Source: Nat Commun. 2026 Jul 20;17:8851. doi: 10.1038/s41467-026-74676-8 (PMC13500464; doi:10.1038/s41467-026-74676-8)
Supplement: Supplementary file 4 [file 41467_2026_74676_MOESM9_ESM.pdf]

nature portfolio

Corresponding author(s): Tiansheng Li; Jonathan Yewdell

Last updated by author(s): May 6, 2026

Reporting Summary

Nature Portfolio wishes to improve the reproducibility of the work that we publish. This form provides structure for consistency and transparency in reporting. For further information on Nature Portfolio policies, see our Editorial Policies and the Editorial Policy Checklist.

Statistics

For all statistical analyses, confirm that the following items are present in the figure legend, table legend, main text, or Methods section.

n/a	Confirmed	
☐	☒	The exact sample size (*n*) for each experimental group/condition, given as a discrete number and unit of measurement
☐	☒	A statement on whether measurements were taken from distinct samples or whether the same sample was measured repeatedly
☐	☒	The statistical test(s) used AND whether they are one- or two-sided<br>*Only common tests should be described solely by name; describe more complex techniques in the Methods section.*
☒	☐	A description of all covariates tested
☐	☒	A description of any assumptions or corrections, such as tests of normality and adjustment for multiple comparisons
☐	☒	A full description of the statistical parameters including central tendency (e.g. means) or other basic estimates (e.g. regression coefficient) AND variation (e.g. standard deviation) or associated estimates of uncertainty (e.g. confidence intervals)
☐	☒	For null hypothesis testing, the test statistic (e.g. *F*, *t*, *r*) with confidence intervals, effect sizes, degrees of freedom and *P* value noted<br>*Give P values as exact values whenever suitable.*
☒	☐	For Bayesian analysis, information on the choice of priors and Markov chain Monte Carlo settings
☒	☐	For hierarchical and complex designs, identification of the appropriate level for tests and full reporting of outcomes
☒	☐	Estimates of effect sizes (e.g. Cohen's *d*, Pearson's *r*), indicating how they were calculated

Our web collection on statistics for biologists contains articles on many of the points above.

Software and code

Policy information about availability of computer code

Data collection	LAS-X was used for confocal microscopy image acquisition; Cytation 5 and Gen5 (v 3.12) were used for high-throughput image acquisition; FACS Diva (v 8.0) was used for flow cytometry data acquisition; and MSD Discovery Workbench 4.0.12 (LSR_4_0_12) was used for proinflammatory cytokine data acquisition.
Data analysis	Microsoft Excel (v 16.8) and GraphPad Prism (v 9.02) were used for data analysis; FlowJo was used for flow cytometry data analysis; LAS-X (v 5.0), Imaris (v 9.0), and Gen5 (v 3.12) were used for image processing and analysis. Phylogenetic analysis was performed using Clustal Omega.

For manuscripts utilizing custom algorithms or software that are central to the research but not yet described in published literature, software must be made available to editors and reviewers. We strongly encourage code deposition in a community repository (e.g. GitHub). See the Nature Portfolio guidelines for submitting code & software for further information.

Data

Policy information about availability of data

All manuscripts must include a data availability statement. This statement should provide the following information, where applicable:

- Accession codes, unique identifiers, or web links for publicly available datasets
- A description of any restrictions on data availability
- For clinical datasets or third party data, please ensure that the statement adheres to our policy

All data relevant to the manuscript are included. Further details and information are available from the corresponding author upon request. Sequences of the spike protein from variants were downloaded from NCBI, with access numbers indicated in the manuscript. The SARS-CoV-2 genome sequences were downloaded from

GISAID (https://gisaid.org/) and Our World in Data (https://ourworldindata.org/covid-vaccinations). The code for plotting the proportion of dominant SARS-CoV-2 variants over time and for calculating the median date of each variant has been deposited on Gitlab(https://gitlab.com/dashboard/snippets).

Research involving human participants, their data, or biological material

Policy information about studies with human participants or human data. See also policy information about sex, gender (identity/presentation), and sexual orientation and race, ethnicity and racism.

Reporting on sex and gender	N/A
Reporting on race, ethnicity, or other socially relevant groupings	N/A
Population characteristics	N/A
Recruitment	N/A
Ethics oversight	N/A

Note that full information on the approval of the study protocol must also be provided in the manuscript.

Field-specific reporting

Please select the one below that is the best fit for your research. If you are not sure, read the appropriate sections before making your selection.

[x] Life sciences [ ] Behavioural & social sciences [ ] Ecological, evolutionary & environmental sciences

For a reference copy of the document with all sections, see nature.com/documents/nr-reporting-summary-flat.pdf

Life sciences study design

All studies must disclose on these points even when the disclosure is negative.

Sample size	All experiments in cell lines were performed independently at least three times. Imaging experiment fields were chosen to ensure representative population capture, and images for analysis were randomly acquired. The sample size for each experiment is indicated in the manuscript, based on previously published studies.
Data exclusions	No data were excluded.
Replication	All data were replicated multiple times, as indicated in the figure legends.
Randomization	Experimental groups were randomly allocated.
Blinding	The investigators were not blinded to allocation during the experiment and outcome assessment, as these experiments were performed by the same investigators.

Reporting for specific materials, systems and methods

We require information from authors about some types of materials, experimental systems and methods used in many studies. Here, indicate whether each material, system or method listed is relevant to your study. If you are not sure if a list item applies to your research, read the appropriate section before selecting a response.

Materials & experimental systems

n/a	Involved in the study	
[ ]	[x]	Antibodies
[ ]	[x]	Eukaryotic cell lines
[x]	[ ]	Palaeontology and archaeology
[ ]	[x]	Animals and other organisms
[x]	[ ]	Clinical data
[ ]	[x]	Dual use research of concern
[x]	[ ]	Plants

Methods

n/a	Involved in the study	
[x]	[ ]	ChIP-seq
[ ]	[x]	Flow cytometry
[x]	[ ]	MRI-based neuroimaging

Antibodies

Antibodies used	The following commercially available antibodies were used in this study. The company, clone, and catalog number are provided below and are publicly available. Antibody dilutions and usage methods are detailed in the manuscript text:

	• Rabbit polyclonal anti-SARS-CoV-2-N (GeneTex, GTX135357, 1:500)<br>• Rabbit monoclonal Alexa Fluor® 647 conjugated anti-Cytokeratin 5 (Cytk5, Abcam, ab193895, 1:200)<br>• Rabbit monoclonal anti-Acetyl-α-tubulin (AcTub, Cell Signaling, 5335, 1:1000)<br>• Mouse monoclonal anti-Mucin 5AC (Muc5A, Invitrogen, MA5-12178, 1:100)<br>• Cross-absorbed goat anti-rabbit Alexa Fluor 750 or 488 (Invitrogen, A21039/A11008, 1:1000)<br>• Cross-absorbed goat anti-mouse Alexa Fluor 488 (Invitrogen, A11029, 1:1000)<br>The antibodies for the neutralization assay can be found in the following references:<br>• Davide Robbiani et al., 2020 (https://www.nature.com/articles/s41586-020-2456-9)<br>• Christopher Barnes et al., 2020 (https://www.sciencedirect.com/science/article/pii/S0092867420307571)<br>• Philip Brouwer et al., 2020 (https://science.sciencemag.org/content/early/2020/06/15/science.abc5902)
Validation	All commercial antibodies have been verified by the manufacturers. The neutralizing antibodies were referenced from the original publications.

Eukaryotic cell lines

Policy information about cell lines and Sex and Gender in Research

Cell line source(s)	Cell lines used in this study included:<br>• Baby hamster kidney fibroblast BHK21 (ATCC; CCL-10)<br>• African green monkey kidney Vero E6 (ATCC; C1008, Clone E6)<br>• Vero (provided by Dr. Nihal Altan-Bonnet, NHLBI)<br>• Human embryonic kidney HEK293FT cells (Thermo Fisher; R70007)<br>• Human lung adenocarcinoma A549 cells (ATCC; CCL-185)<br>• Calu-3 cells (ATCC; HTB-55)<br>• Human colorectal adenocarcinoma Caco-2 cells (ATCC; HTB-37)<br>• BHK21 cells stably expressing human ACE2 (BHK21-ACE2, made in the lab)<br>• A549 cells stably expressing human ACE2 (A549-ACE2, made in the lab)<br>• Vero E6 cells stably expressing human TMPRSS2 (Vero E6-TMPRSS2, made in the lab)<br>• 293FT cells stably expressing T7 polymerase and VSVG (293FT-VSVG-T7pol) was made in the lab. Vero E6 cells stably expressing human ACE2 and TMPRSS2 (Vero E6-AT2) were obtained from BEI (NR-54970). A549 cells stably expressing human ACE2 and TMPRSS2 (ACE2plusC3, ATCC; CRL-3560) were provided by Dr. Ching-Wen Chang (University of Massachusetts Chan Medical School).
Authentication	All stable cell lines generated using the Sleeping Beauty system were functionally validated or confirmed by protein expression using flow cytometry.
Mycoplasma contamination	Cells were regularly tested and found negative for Mycoplasma.
Commonly misidentified lines<br>(See ICLAC register)	No commonly misidentified cell lines were used.

Animals and other research organisms

Policy information about studies involving animals; ARRIVE guidelines recommended for reporting animal research, and Sex and Gender in Research

Laboratory animals	B6.Cg-Tg(K18-ACE2)2Prlmn/J from The Jackson Laboratory(Strain #:034860).
Wild animals	No wild animals were used in this study.
Reporting on sex	Male and female mice were considered in this study without discrimination. Male and female mice were considered in this study without discrimination.
Field-collected samples	This study did not involve field-collected samples.
Ethics oversight	The mice were housed, and experiments were performed according to the animal study protocols approved by the NIH (Animal Study Proposal: LVD-5E, LVD-11E) and the FDA White Oak Animal Program Animal Care and Use Committee.

Note that full information on the approval of the study protocol must also be provided in the manuscript.

Plants

Seed stocks	No plants were used.
Novel plant genotypes	No plants were used.
Authentication	No plants were used.

Flow Cytometry

Plots

Confirm that:

- [x] The axis labels state the marker and fluorochrome used (e.g. CD4-FITC).
- [x] The axis scales are clearly visible. Include numbers along axes only for bottom left plot of group (a 'group' is an analysis of identical markers).
- [x] All plots are contour plots with outliers or pseudocolor plots.
- [x] A numerical value for number of cells or percentage (with statistics) is provided.

Methodology

Sample preparation	Infected cell were lifted with trypsin and transferred into an Eppendrof tube or 96-well plate with 4% PFA and fixed for 20min. Cells were washed three times with PBS and pelleted at 300g for 5min. Cells were resuspended in PBS and analyzed on BD LSRFortessa flow cytometer.
Instrument	BD LSRFortessa flow cytometer
Software	FACS Diva; FlowJo
Cell population abundance	Flow cytometry was done on cell lines infected with rVSV-S or rSARS2 with GFP or RFP. And GFP or RFP level was analyzed, and the positive level was generally high (>5%).
Gating strategy	Cells were gated with FSC-H and SSC-H. Single cells were gated with FSC-H and FSC-A. Then GFP or RFP positive cells were gated based on control groups.

- [ ] Tick this box to confirm that a figure exemplifying the gating strategy is provided in the Supplementary Information.
